# HMGB2 Deficiency Mitigates Abdominal Aortic Aneurysm by Suppressing Ang-II-Caused Ferroptosis and Inflammation via NF-*κβ* Pathway

**DOI:** 10.1155/2023/2157355

**Published:** 2023-12-19

**Authors:** Hao Wu, Legao Chen, Kaiping Lu, Yi Liu, Weiqin Lu, Jinsong Jiang, Chao Weng

**Affiliations:** Department of Vascular Surgery, Zhejiang Provincial People's Hospital (Affiliated People's Hospital), Hangzhou Medical College, 158 Shangtang Road, Hangzhou, Zhejiang, China

## Abstract

**Background:**

Ferroptosis is a new form of cell death, which is closely related to the occurrence of many diseases. Our work focused on the mechanism by which HMGB2 regulate ferroptosis and inflammation in abdominal aortic aneurysm (AAA).

**Methods:**

Reverse transcription–quantitative polymerase chain reaction and western blot were utilized to assess HMGB2 levels. CCK-8 and flow cytometry assays were utilized to measure cell viability and apoptosis. We detected reactive oxygen species generation, Fe^2+^ level, and ferroptosis-related protein levels in Ang-II-treated VSMCs, which were typical characteristics of ferroptosis. Finally, the mice model of AAA was established to verify the function of HMGB2 in vivo.

**Results:**

Increased HMGB2 level was observed in Ang-II-treated VSMCs and Ang-II-induced mice model. HMGB2 depletion accelerated viability and impeded apoptosis in Ang-II-irritatived VSMCs. Moreover, HMGB2 deficiency neutralized the increase of ROS in VSMCs caused by Ang-II. HMGB2 silencing considerably weakened Ang-II-caused VSMC ferroptosis, as revealed by the decrease of Fe^2+^ level and ACSL4 and COX2 levels and the increase in GPX4 and FTH1 levels. Furthermore, the mitigation effects of shHMGB2 on Ang-II-induced VSMC damage could be counteracted by erastin, a ferroptosis agonist. Mechanically, HMGB2 depletion inactivated the NF-*κβ* signaling in Ang-II-treated VSMCs.

**Conclusions:**

Our work demonstrated that inhibition of HMGB2-regulated ferroptosis and inflammation to protect against AAA via NF-*κβ* signaling, suggesting that HMGB2 may be a potent therapeutic agent for AAA.

## 1. Introduction

Abdominal aortic aneurysm (AAA) is a fatal vascular disease with a mortality rate of nearly 100% after AAA rupture [1]. Most patients are at serious risk of vascular rupture at the time of diagnosis because of its insidious progression and the difficulty of early diagnosis [2]. One of the notable histopathological features of AAA is severe degeneration of elastic mediators, substantial loss of elastin, and vascular smooth muscle cells (VSMCs), resulting in a reduced density of VSMC, weakened aortic wall, and possible rupture of artery wall [3]. Due to the lack of effective drug therapy, the clinical treatment of AAA is limited to surgical intervention [4]. It is pivotal to explore the molecular mechanism of AAA development to ascertain new therapeutic targets for AAA.

Ferroptosis is a specific form of regulatory cell death characterized by accumulation of iron and overproduction of lipid reactive oxygen species (ROS) [5]. Numerous reports have suggested that ferroptosis is associated with multiple cardiovascular diseases, such as cardiac ischemia–reperfusion injury [6], acute myocardial infarction [7], atherosclerosis [8], and heart failure [9]. Moreover, published studies suggested that SCD, PRXD1, and IL-6 may influence the progress of AAA by the modulating ferroptosis [10]. The exact mechanism that regulated ferroptosis and affected AAA has not yet been studied.

HMGB protein is a DNA binding protein that regulates DNA transcription, replication, and repair [11]. The HMGB family, including HMGB1–4, acts as pro-inflammatory mediators in a variety of diseases [12]. Of the four HMGB members, HMGB1 affects AAA progression by regulating the necroptosis in Ang-II-induced AAA model in APOE^−/−^ mice [13]. The amino acid sequences of HMGB1 and HMGB2 are highly similar [14]. However, the role of HMGB2 in the pathogenesis of AAA remains unknown.

In this work, we used Ang-II-induced VSMC model and Ang-II-stimulated Apoe^−/−^ mouse model to explore the potential mechanism of HMGB2 in AAA development. Our findings may offer new insights for the future application of HMGB2 in AAA.

## 2. Methods

### 2.1. Tissue Samples

AAA tissues were collected from 48 AAA patients who underwent surgery in our hospital. Normal abdominal aortic tissue came from age- and sex-matched organ donors without aortic disease. Tissue samples were cut into small segments in liquid nitrogen and further stored at −80°C. All patients signed informed consent. This study was approved by the Ethics Committee of the Zhejiang Provincial People's Hospital (Affiliated People's Hospital).

### 2.2. Cell Culture and Treatment

VSMCs were purchased from Procell Co. (Wuhan, China). VSMCs were cultured in DMEM containing FBS (10%), glucose (4.5 g), penicillin (100 U/mL), and streptomycin (100 *μ*g/mL) and kept in a humid environment with 5% CO_2_, at 37°C. The passage five cells were treated with Ang-II (1 *μ*mol/L) and cultured in serum-free medium for 24 hr with 70%–80% confluence.

shRNA against S100A4 were synthesized by Vigene Bioscience (Jinan, Shandong, China). VSMC was inoculated into 6-well plates at 50%–70% concentration and cells were starved for 24 hr with DMEM. The cells were transfected with shS100A4 via Lipofectamine 2000 (Invitrogen). After incubation for 12 hr, the cells were incubated in DMEMs containing FBS and penicillin/streptomycin. After 24-hr growth, the cells were treated with Ang-II (1 *μ*mol/L) for subsequent tests.

### 2.3. Reverse Transcription-Quantitative Polymerase Chain Reaction (RT-qPCR)

Total RNA was extracted from VSMCs with an RNA extraction kit (Promega) and reversely transcribed into complementary DNA (cDNA). Next, qPCR was performed in ABI StepOnePlus™ Real-Time PCR Systems (Thermo Fisher Scientific) using SYBR® Premix Ex Taq™ (Tli RNaseH Plus). Relative gene levels were calculated by 2^−*ΔΔ*Ct^ method and normalized to GAPDH.

### 2.4. CCK-8

VSMCs were inoculated in 96-well plates (5 × 10^3^/well) and cultured until the specified time. After adding CCK-8 reagent (10 *μ*L/well), VSMCs were cultured for 2 hr. Finally, the optical density (OD) was assessed with a microplate reader (Thermo Scientific, USA) at 450 nm.

### 2.5. Flow Cytometry

VSMCs (1 × 10^5^) were resuspended in 1 × binding buffer, stained with FITC-conjugated Annexin V and PI (BD Pharmingen) for 15 min in darkness. Finally, a flow cytometer (BD Biosciences) was used to analyze VSMC apoptosis.

### 2.6. Animal Model

Male ApoE^−/−^ mice (8 weeks) were acquired from Beijing Weishanglide Animal Experimental Center. All the mice were placed in specific pathogen-free environments and kept on a 12-hr cycle of light and darkness, with free food and water.

In vivo experiments, the mice were randomly divided into four groups (*n* = 20). For Sham group, mice were injected with normal saline. For Ang-II group, mice were injected with 1,000 ng·kg^−1^·min^−1^ Ang-II. For Ang-II + shNC group, mice were injected with shNC via tail vein for 4 weeks and injected with 1,000 ng·kg^−1^·min^−1^ Ang-II. For Ang-II + shHMGB2 group, mice were injected with shHMGB2 via tail vein for 4 weeks and injected with 1,000 ng·kg^−1^·min^−1^ Ang-II. After 4 weeks of drug treatment, all mice were killed with pentobarbital and samples were collected for the further histological studies.

### 2.7. Histology of Mice

The mice were sacrificed, abdominal aorta was taken, fixed for 12 hr, paraffin embedded, and a cross-section of 6 *μ*m was prepared. Paraffin sections were stained with hematoxylin and eosin (HE) and Verhoeff-van Gieson (VVG).

### 2.8. Immunofluorescence Staining

The slides containing frozen sections of aorta were fixed in 4% paraformaldehyde for 15 min and rinsed three times with PBS. The sections were incubated overnight at 4°C and the following primary antibody was applied: anti-*α*-SMA and anti-HMGB2. The slices were then incubated in secondary antibody for 60 min. Sections were rinsed three times with PBS and stained with DAPI. Slides of secondary antibody culture were used as negative control.

### 2.9. Western Blotting

Total proteins in cells or tissues were lysed with RIPA lysate (Invitrogen). After quantifying the protein concentration, the protein was isolated by 10% SDS-PAGE and then transferred to PVDF membrane. After being blocked by 5% nonfat milk and mixed with primary antibodies against HMGB2, GPX4, FTH1, ACSL4, COX2, and GAPDH and secondary antibodies. Finally, the protein bands were visualized with the ECL system (BIO-RAD, USA).

### 2.10. DHE Staining

To analysis ROS level in mice abdominal aorta, frozen aortas were incubated with DHE (5 mM) for 30 min. After washing with PBS, the tissue was covered with DAPI anti-fading reagent and imaged under a fluorescence microscope. To analysis ROS level in cells, different treatment groups of cells were incubated with DHE (5 mM) for 30 min and DAPI (10 mM) for 5 min. The images were captured quickly under a fluorescence microscope.

### 2.11. Transmission Electron Microscope Assay

The VSMCs were collected and added to 0.1 M phosphate-buffered brine (PBS, pH 7.4) with 2.5% glutaraldehyde and fixed at room temperature for 24 hr. The cells were then fixed in 2% osmium tetroxide in PBS for 1 hr. Next, samples were dehydrated with an ethanol solution (50%–100%). Subsequently, the sample was embedded and sliced into ultrathin slices (60 nm). Finally, the results were observed using H-7500 TEM (Hitachi, Japan).

### 2.12. ELISA Assay

The concentration of IL-6 and IL-1*β* was assessed using ELISA Kit (R&D System) as per the supplier's instructions.

### 2.13. Ferrous Iron Assay

The levels of Fe^2+^ measured by using the Iron Assay Kit. The kits were obtained from Abcam (Cambridge, USA).

### 2.14. Statistical Analyses

All data were presented as the mean ± SD and executed using GraphPad Prism 7. Statistical differences were operated by Student's *t*-test or one-way ANOVA. *P* < 0.05 was defined as significant.

## 3. Results

### 3.1. HMGB2 Was Enhanced in AAA

**Figure 1:**
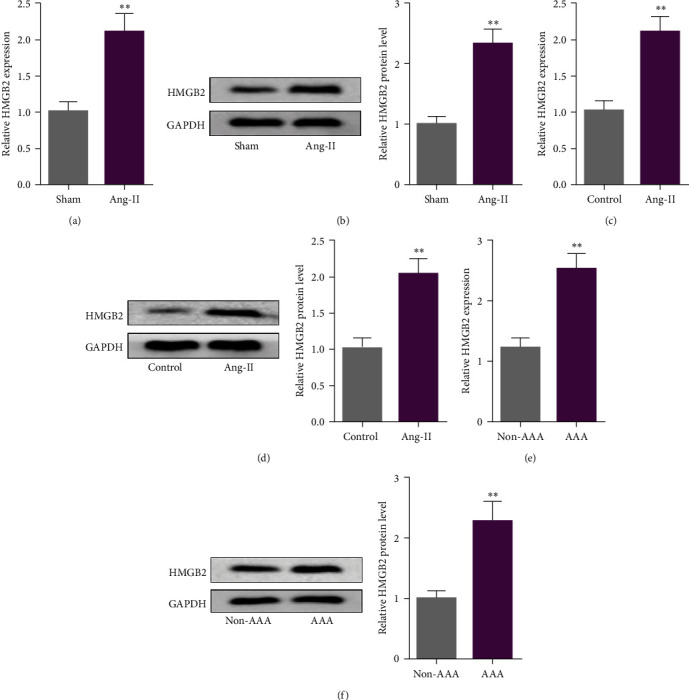
HMGB2 was enhanced in AAA. The expression of HMGB2 was assessed by RT-qPCR and western blot in Ang-II perfused aortas in Apoe^−/−^ mice (a and b). RT-qPCR and western blot were utilized to measure HMGB2 level in Ang-stimulated VSMCs (c and d). The level of HMGB2 was measured in human AAA tissues and normal aortas (e and f).

To determine whether HMGB2 is related to the progression of AAA, we evaluated its level in Apoe^−/−^ mice infused with Ang-II, a recognized experimental AAA model [15]. High level of HMGB2 was found in AAA group relative to Sham group (Figures 1(a) and 1(b)). VSMCs play a key role in maintaining vascular homeostasis, and their dysfunction is a vital response to AAA [16]. We established an in vitro AAA cell model by stimulating VSMCs with Ang-II. As expected, HMGB2 expression was elevated in Ang-stimulated VSMCs (Figures 1(c) and 1(d)). Consistently, HMGB2 mRNA and protein level were increased in human AAA tissues compared with normal aorta from the organ donor (Figures 1(e) and 1(f)). Hence, we assumed that HMGB2 played a contributing role in AAA development.

### 3.2. HMGB2 Deficiency Enhanced Viability and Inhibited Apoptosis in Ang-II-Treated VSMCs

**Figure 2:**
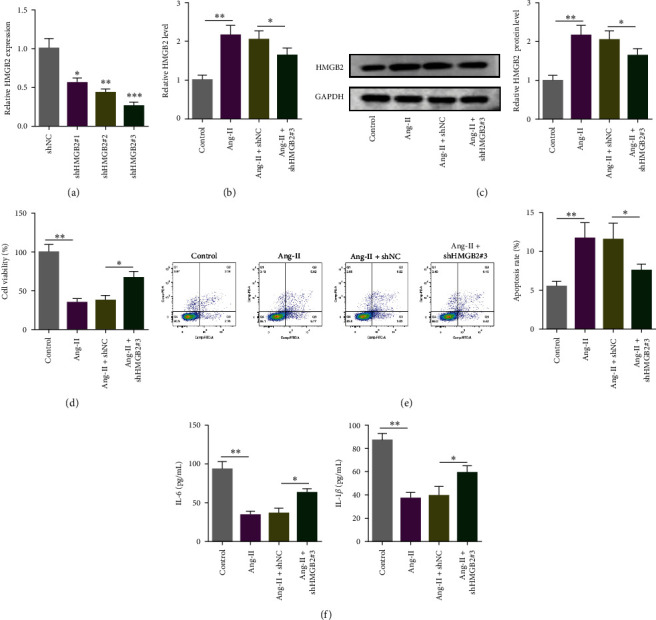
HMGB2 deficiency enhanced viability and inhibited apoptosis in Ang-II-treated VSMCs. shHMGB2#1, shHMGB2#2, or shHMGB2#3 were transfected into Ang-II-treated VSMCs to detect the expression of HMGB2 (a). The Ang-II-induced VSMCs were treated with shNC or shHMGB2#3. HMGB2 mRNA and protein level were detected (b and c). CCK-8 and flow cytometry assays were performed to assess Ang-II-treated VSMC viability and apoptosis (d and e). ELISA assay detected IL-6 and IL-1*β* in Ang-II-treated VSMCs (f).

To verify the functions of HMGB2 in AAA, three HMGB2 shRNAs were established. Transfection of shHMGB2#1, shHMGB2#2, or shHMGB2#3 distinctly inhibited HMGB2 level in Ang-II-challenged VSMCs. sh100A4#3 has the highest knockout efficiency and was used for subsequent experiments (Figure 2(a)). Next, VSMCs were assigned to control, Ang-II, Ang-II + shNC, and Ang-II + shHMGB2#3 groups. HMGB2 level was elevated in Ang-II group, which was partially offset by shHMGB2#3 (Figures 2(b) and 2(c)). Additionally, shHMGB2#3 transfection markedly enhanced Ang-II-impeditive VSMCs viability (Figure 2(d)). The stimulative apoptosis of Ang-II-induced in VSMCs was suppressed by shHMGB2#3 (Figure 2(e)). Furthermore, IL-6 and IL-1*β* levels were significantly elevated in Ang-II induction, as shown in Figure 2(f), and shHMGB2#3 significantly reversed these changes. Based on these findings, HMGB2 silencing reversed Ang-II-mediated VSMC viability, apoptosis, and inflammation.

### 3.3. HMGB2 Depletion Suppressed Ang-II-Caused Ferroptosis in VSMCs

**Figure 3:**
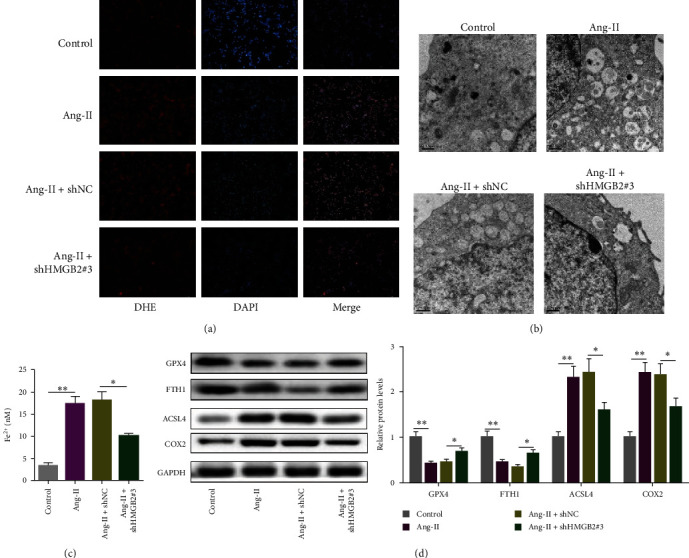
HMGB2 depletion suppressed Ang-II-caused ferroptosis in VSMCs. DHE staining was used to detect ROS generation (a). Mitochondrial status was observed under TEM in Ang-II-induced VSMCs (b). Fe^2+^ level, GPX4, FTH1, ACSL4, and COX2 level were detected in Ang-II-induced VSMC transfected with shNC, or shHMGB2#3 (c and d).

The accumulation of intracellular ROS is one of the characteristic manifestations of ferroptosis [17]. As illustrated in Figure 3(a), Ang-II caused a substantial increase in ROS generation, which was reversed by shHMGB2 transfection. Moreover, Ang-II resulted in a distinct decrease in mitochondrial volume and an increase in membrane density. The mitochondrial crista decreased or disappeared under transmission electron microscopy. This is a typical feature of ferroptosis. After transfection of shHMGB2, the change of mitochondrial morphology was partially reversed (Figure 3(b)). Meanwhile, iron accumulation, ferroptosis inhibitors (GPX4 and FTH1), and ferroptosis promoters (ACSL4 and COX2) were assessed. It was shown that Ang-II decreased GPX4 and FTH1 protein levels, and enhanced Fe^2+^ level and ACSL4, and COX2 levels in VSMCs; however, HMGB2 depletion eliminated such effects, indicating the repressive effect of shHMGB2 on Ang-II-induced ferroptosis in VSMCs (Figures 3(c) and 3(d)). Taken together, deficiency of HMGB2 repressed ferroptosis in Ang-II-challenged VSMCs.

### 3.4. Erastin Eliminated the Repressive Effect of shHMGB2 against Ang-II-Induced Ferroptosis in VSMCs

**Figure 4:**
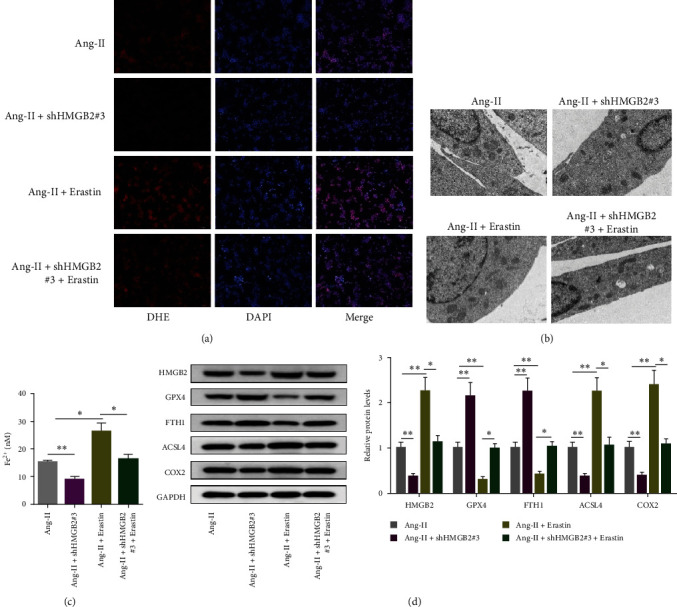
Erastin eliminated the repressive effect of shHMGB2 against Ang-II-induced ferroptosis in VSMCs. VSMCs were assigned to Ang-II, Ang-II + shHMGB2#3, Ang-II + shHMGB2#3, and Ang-II + shHMGB2#3 + Erastin groups. DHE staining was performed to detect ROS generation (a). Mitochondrial status was observed under TEM in Ang-II-induced VSMCs (b). Fe^2+^ level, GPX4, FTH1, ACSL4, and COX2 level were measured (c and d).

Using ferroptosis agonist (erastin, 10 *μ*M), we further confirmed that VSMCs underwent ferroptosis. In Figure 4(a), erastin eliminated shHMGB2-mediated suppression of ROS generation in Ang-II-triggered VSMCs. The improved mitochondrial morphology of shHMGB2 was destroyed by erastin (Figure 4(b)). Moreover, HMGB2 depletion inhibited iron content, upregulated GPX4 and FTH1 protein levels, and downregulated HMGB2, ACSL4, and COX2 protein levels, which was neutralized by erastin (Figures 4(c) and 4(d)). The above results indicated that HMGB2 knockdown inhibited AAA by inhibiting oxidative stress and ferroptosis.

### 3.5. HMGB2 Knockdown Alleviates Ang-II-Caused Ferroptosis by Activating the NF-*κβ* Signaling

**Figure 5:**
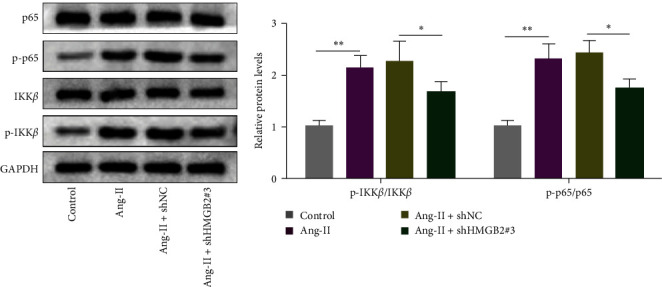
HMGB2 knockdown alleviated Ang-II-caused ferroptosis by activating the NF-*κβ* signaling. VSMCs were assigned to Ang-II, Ang-II + shHMGB2#3, Ang-II + shHMGB2#3, and Ang-II + shHMGB2#3 + Erastin groups. The protein level of IKK*β*, p-IKK*β*, p65, and p-p65 were measured by western blot.

NF-*κβ* activation has been shown to be a catalyst for oxidative stress and ferroptosis in a variety of diseases [18]. Moreover, NF-*κβ* pathway activation has been previously reported in patients with AAA [19]. We hypothesize that HMGB2 may influence AAA progression through NF-*κβ* pathway. To elucidate the role of the NF-*κβ* pathway in the protective effect of shHMGB2 against Ang-II-induced VSMCs, we evaluated the levels of key proteins in the NF-*κβ* pathway (IK*β*, p-IKK*β*, p65, and p-p65). As indicated in Figure 5, Ang-II stimulation enhanced p-IKK*β* and p-p65 levels in VSMCs, shHMGB2 counteracted the effects of Ang-II stimulation. Overall, HMGB2 knockdown alleviated Ang-II-triggered inflammation and ferroptosis by inactivating the NF-*κβ* pathway.

### 3.6. Silencing of HMGB2 Suppressed Ang-II-Stimulated AAA Formation and Ferroptosis In Vivo

**Figure 6:**
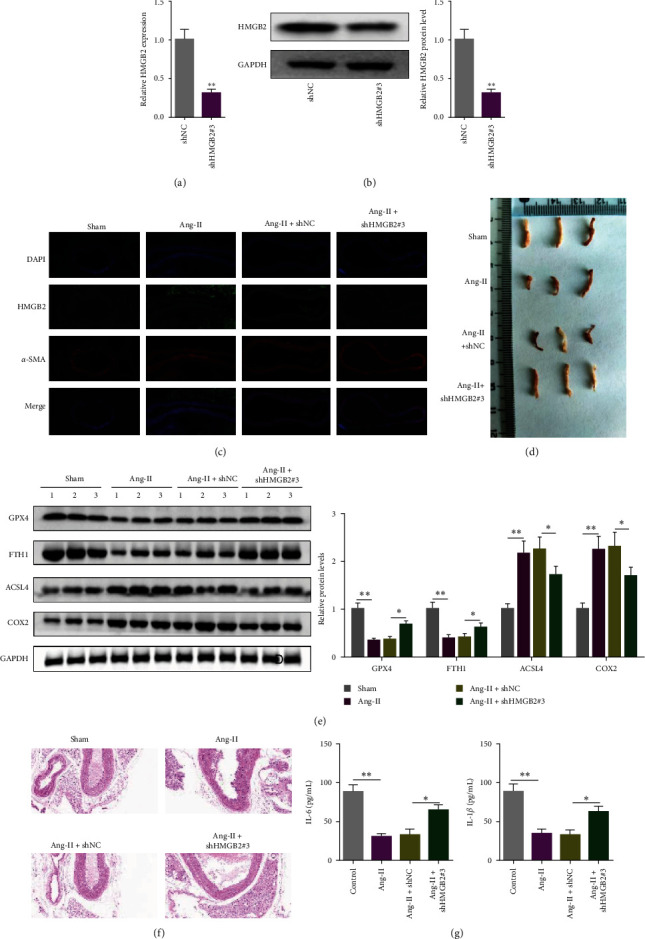
Silencing of HMGB2 suppressed Ang-II-stimulated AAA formation and ferroptosis in vivo. Mice were injected with Ang-II to establish an AAA model. RT-qPCR and western blot assays were used to measure HMGB2 level (a and b). Immunofluorescence staining was utilized to measure HMGB2 and *α*-SMA expression (c). The representative images of aorta in Sham, Ang-II, Ang-II + shNC, and Ang-II + shHMGB2#3 groups (d). GPX4, FTH1, ACSL4, and COX2 protein levels were measured by western blot (e). Typical images showing HE staining for aortic cross-sections (f). ELISA assay was performed to detect IL-6 and IL-1*β* level (g).

To further investigate the effects of HMGB2 on the formation of AAA, we first knockdown HMGB2 and verified by RT-qPCR and western blot (Figures 6(a) and 6(b)). IF assay showed that Ang-II-treated mice showed a prominent reduction in *α*-smooth muscle actin (*α*-SMA) and marked increase in HMGB2, and these reactions were partially reversed by shHMGB2 (Figure 6(c)). Moreover, after Ang-II perfusion, large AAAs were evident in Apoe^−/−^ mice, which were neutralized by shHMGB2. Transfection of shHMGB2 alleviated Ang-II-induced intramural thrombus and severe aortic dilatation in mice (Figure 6(d)). Additionally, it was shown that Ang-II decreased GPX4 and FTH1 protein levels, and enhanced ACSL4 and COX2 protein levels in vivo, which was partly eliminated by HMGB2 depletion (Figure 6(e)). In Ang-II group, aortic wall thrombosis in AAA caused severe aortic dilation, which was relieved after shHMGB2 injection (Figure 6(f)). Furthermore, HMGB2 deletion reversed the stimulative effects of Ang-II on IL-6 and IL-1*β* in Apoe^−/−^ mice (Figure 6(g)). The results of in vivo experiments further confirmed the important role of HMGB2 in the formation of AAA.

## 4. Discussion

AAA is a degenerative disease that irreversibly affects human health and is closely associated with a high-mortality rate after aortic rupture [20]. Currently, there are no effective drugs to prevent and treat AAA [21]. Previous studies have shown that regulating levels of genes associated with inflammation is a promising therapeutic strategy for controlling progression [22]. This research first revealed the vital role of the pro-inflammatory factor HMGB2 in AAA by regulating inflammation and ferroptosis in vitro and in vivo.

HMGB protein is the second most abundant protein in humans and plays a global genomic function in establishing active or inactive chromatin domains [23]. The HMGB protein family has been implicated in the development of a variety of inflammatory diseases [24]. According to the previous studies, HMGB2-accelerated myocardial ischemia injury through ROS-mediated apoptosis, abnormal autophagy, and inflammatory response [25]. Moreover, HMGB2 has recently been identified as a key driver of several vascular diseases, including atherosclerosis [26] and coronary artery in-stent restenosis [14]. HMGB1 has been shown to facilitate AAA progression. The biological characteristics of HMGB1 are difficult to distinguish from HMGB2. However, the potential role of HMGB2 in AAA has not been studied until now. Our study, through in vivo and in vitro experiments, demonstrates the role of HMGB2 in promoting AAA, further highlighting its harmful role in the different vascular diseases.

In order to better understand the key role of HMGB2 in AAA formation, we established cell models and mice models to affirm the role of HMGB2 in promoting AAA formation. In Ang-II-induced VSMCs, cell viability was inhibited and apoptosis and inflammation was enhanced, and the effect of Ang-II induction was partially reversed after transfection with shHMGB2. Previous reports have indicated the catalytic role of ROS in the formation of AAA [27]. High-ROS levels can induce cell death through oxidative damage to proteins, DNA, and cell structures [28]. Liu et al. [25] pointed that HMGB2 increased ROS production in ischemic myocardium. In this study, Ang-II treated remarkably increased ROS production in VSMCs, which was partially reversed by the HMGB2 silencing. Abnormal iron metabolism and ROS production are signs of ferroptosis [29]. Ferroptosis has been shown to be essential for the prevention of various vascular diseases [30]. Therefore, targeting VSMC ferroptosis might be an effective treatment for alleviating AAA. Here, we found that Ang-II-induced VSMC ferroptosis through observing the mitochondrial morphology in TEM images. Moreover, iron level and ferroptosis-related proteins (GPX4, FTH1, ACSL4, and COX2) were measured to further investigate Ang-II-induced ferroptosis in VSMCs. Results revealed that Ang-II increased iron content, inhibited GPX4 and FTH1 protein levels, and upregulated ACSL4 and COX2 protein levels in VSMCs. These data showed that Ang-II-caused VSMC ferroptosis. Moreover, Ang-II-induced ferroptosis could be effectively reversed by shHMGB2. NF-*κβ* signaling is one of the classical inflammatory pathways, which plays a crucial role in the progression of many diseases [31]. Inhibition of NF-*κβ* signaling pathway could alleviate inflammatory symptoms and thus affect the progression of diseases [32]. Moreover, previous studies have shown that NF-*κβ* pathway activation promoted ferroptosis in the glioblastoma cells [33]. In this study, the levels of key proteins of the NF-*κβ* signaling were assessed. VSMCs transfected with shHMGB2 markedly alleviated the activation of the NF-*κβ* pathway caused by Ang-II. Therefore, we indicated that HMGB2 knockdown alleviated Ang-II-triggered inflammation and ferroptosis by inactivating the NF-*κβ* signaling pathway. In the future, more experiments will be conducted to verify the effect of HMGB2 on AAA progression by regulating the NF-*κβ* pathway.

## 5. Conclusion

In summary, our findings suggested that depletion of HMGB2 promoted viability and restrained apoptosis, inflammation, and ferroptosis through inactivating the NF-*κβ* pathway in Ang-II-treated VSMCs and Ang-II-stimulated Apoe^−/−^ mice. HMGB2 silencing may be a prospective therapeutic agent for AAA.

## Data Availability

The datasets used and/or analyzed during the current study are available from the corresponding author on reasonable request.
